# Delineating Molecular Subtypes through Gene Set Variation Analysis Confers Therapeutic and Prognostic Capability in Gastric Cancer

**DOI:** 10.1155/2022/5415758

**Published:** 2022-07-15

**Authors:** Yuzhang Zhu, Ting Sun, Lei Zhang, Faming Fei, Yi Bao, Zhenzhen Gao

**Affiliations:** ^1^Department of Clinical Oncology, The Second Affiliated Hospital of Jiaxing University, Jiaxing, China; ^2^Hospice Care Center of Jiaxing, The Second Hospital, Jiaxing, China

## Abstract

To claim the features of nontumor tissue in gastric cancer patients, especially in those who have undergone gastrectomy, and to identify the molecular subtypes, we collected the immunogenic and hallmark gene sets from gene set enrichment analysis. The activity changes of these gene sets between tumor (375) and nontumor (32) tissues acquired from the Cancer Genome Atlas (TCGA-STAD) were calculated, and the novel molecular subtypes were delineated. Subsequently, prognostic gene sets were determined using least absolute shrinkage and selection operator (lasso) regression prognostic method. In addition, functional analysis was conducted. Totally, three subtypes were constructed in the present study, and there were differences in survival among three groups. Functional analysis showed genes from normal gene set were related to cell adhesion, and genes from tumor gene set were associated with focal adhesion, PI3K-Akt signaling pathway, regulation of actin cytoskeleton, and VEGF signaling pathway. Our study created lasting value beyond molecular subtypes and underscored the significance of normal tissues in gastric cancer development, which drawn a novel prognostic model for gastric treatment.

## 1. Introduction

Gastric cancer ranks the fifth most common malignancy worldwide and remains the dominant cause of cancer-related deaths in China [1]. The age-adjusted incidence rate (21.7 per 100,000 reported in 2019) in China is significantly higher than that in most developed countries, reminding us of the urgency of gastric cancer prevention and treatment in the future [1, 2]. A large proportion of patients present with distant metastatic lymph nodes at diagnosis. Overall, 5-fluorouracil (5-FU) (fluoropyrimidines)-based or platinum-based chemotherapy is still the mainstay of treatment in metastatic settings [3, 4]. Since the completion of the ToGA study in 2014, the combination of human epidermal growth factor receptor 2 (HER2) inhibitors and chemotherapy has become the standard treatment for advanced gastric cancer patients with HER2 amplification, prolonging the overall survival (OS) to 13.8 months [5–7]. In those patients with HER2-negative gastric cancer, vascular endothelial growth factor (VEGF)-mediated angiogenesis is necessary for pathogenesis [8–10]. Therefore, various antiangiogenic drugs, apatinib, bevacizumab, and ramucirumab have been approved as a standard treatment for prolonging overall survival in subsequent treatment setting [11]. However, the increasement of patients' OS is commonly companied by relapse in tumor growth after several weeks or months, which elicits resistance to anti-VEGF molecules, and the resistance confers the cancer cells a resistant phenotype [12]. Given the essential role of angiogenesis in tumor recurrence, a fulfilled mechanism of its modulation is necessary. Some microRNAs (miRNAs) have been highlighted to be related with angiogenesis. MiR-135a inhibits tumor growth, migration, invasion, and angiogenesis by targeting the focal adhesion kinase pathway, modulating VEGF signaling. In parallel, a link to angiogenesis is implied by the fact that Sp1 overexpression is associated with the upregulation of VEGF in gastric cancer, and two targets of miR-218 have been identified: Angiopoietin-2 and ROBO1 (roundabout guidance receptor 1), and their downregulation result in a reduction of tumor proliferation, invasion, and angiogenesis [12]. These studies indicated potential value of miRNAs to be biomarkers for antiangiogenesis. Aside from this, considering the crosstalk between angiogenesis and immune cells might be involved with resistance to antiangiogenesis therapy, and the association of multiple antiangiogenic molecules or a combination of antiangiogenic drugs with other treatment regimens has been indicated as alternative therapeutic strategies to overcome resistance to antiangiogenic therapies [5, 13]. All the studies above moved the field of chemotherapy toward targeted therapy based on molecular profiling.

The current molecular subtypes were built on multiple dimensions encompassing transcriptome profiling [14, 15], somatic variation [16, 17], simple nucleotide variation [18], DNA methylation [19], anatomic position [20], and histology, mainly acquired from tumor tissues or cell lines, which ignored the features of nontumor tissue in gastric cancer. Additionally, more attention has been paid to the effect of singular pathways or specific molecules in patients with gastric cancer rather than comprehensive pathway activity analysis. Therefore, additional molecular profiling is urgently needed for clinical practice owing to the intrinsic heterogeneity of gastric cancer.

In this study, we outlined a novel stratification method including systematic pathway activity changes of gene sets in both tumor tissues and nontumor tissues and provided a more accurate prognostic model based on least absolute shrinkage and selection operator (LASSO) regression for patients with gastric cancer, which might help to improve patient outcomes, to offer suggestions after gastrectomy, and to facilitate personalized therapy in the nearer future.

## 2. Materials and Methods

### 2.1. Data Resource and Extraction

RNA expression profiling of stomach adenocarcinoma and adjacent normal tissues from the Cancer Genome Atlas (错误!超链接引用无效。TCGA, https://portal.gdc.cancer.gov/) database (TCGA-STAD) were downloaded as the test group. Gene expression data collected from the Gene Expression Omnibus (GEO) database (GSE84437) were used as the validation group. The corresponding clinical data were downloaded from TCGA-GDC. Immunogenic and hallmark gene sets comprising those gene sets, which represented cell states and perturbations within the immune system, were acquired from gene set enrichment analysis (GSEA, https://www.gsea-msigdb.org/gsea/msigdb/).

### 2.2. Conduction of Gene Set Variation Analysis and Distinguishing Subtypes

Gene set enrichment in different samples was obtained by gene set variation analysis (GSVA, https://bioconductor.org/), described as noted. Thereafter, Nbclust, factoextra, and cancersubtypes packages were used to acquire the optimal classification value and to ascertain subtypes.

### 2.3. Establishment of the LASSO Regression Model and Survival Analysis

First, a Cox regression model was used to estimate the gene sets related to the prognosis of patients with gastric cancer, and LASSO regression was performed to calculate the cutoff value and to detect potential normal and tumor gene sets. Furthermore, a survival analysis was performed to determine the prognostic LASSO model.

### 2.4. Functional Enrichment and Protein-Protein Interaction Analysis

Gene ontology (GO)-enrichment analysis and Kyoto Encyclopedia of Genes and Genomes (KEGG) enrichment analysis were used to determine the molecular functions, biological processes, and cellular pathways of the model genes involved in the model gene sets. The Search Tool for the Retrieval of Interacting Genes/Proteins (STRING) database (https://string-db.org) was used to map the proteins of the model genes, and the networks were visualized using Cytoscape (version 3.9.0).

### 2.5. Statistical Analyses

All analyses were performed in *R* (version 4.1.2). *R* packages comprising *GSEABase*, *GSVA*, *limma*, *pheatmap*, *ggplot2*, *VennDiagram*, *survival*, and *glmnet* were used. Statistical significance was set at *P* < 0.05.

## 3. Results

### 3.1. Identification of Discrepant Pathway Activity between Gastric Cancer and Adjacent Normal Tissues

**Figure 1:**
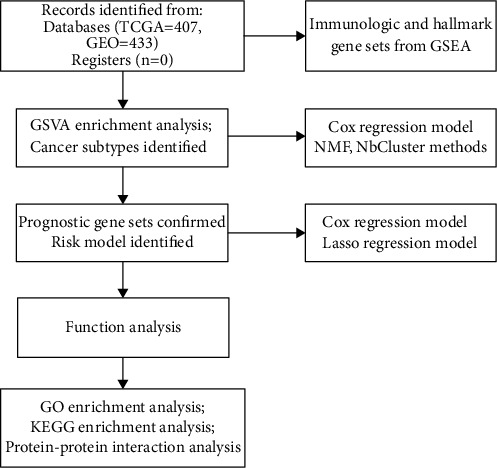
Flow diagram of the study.

**Figure 2:**
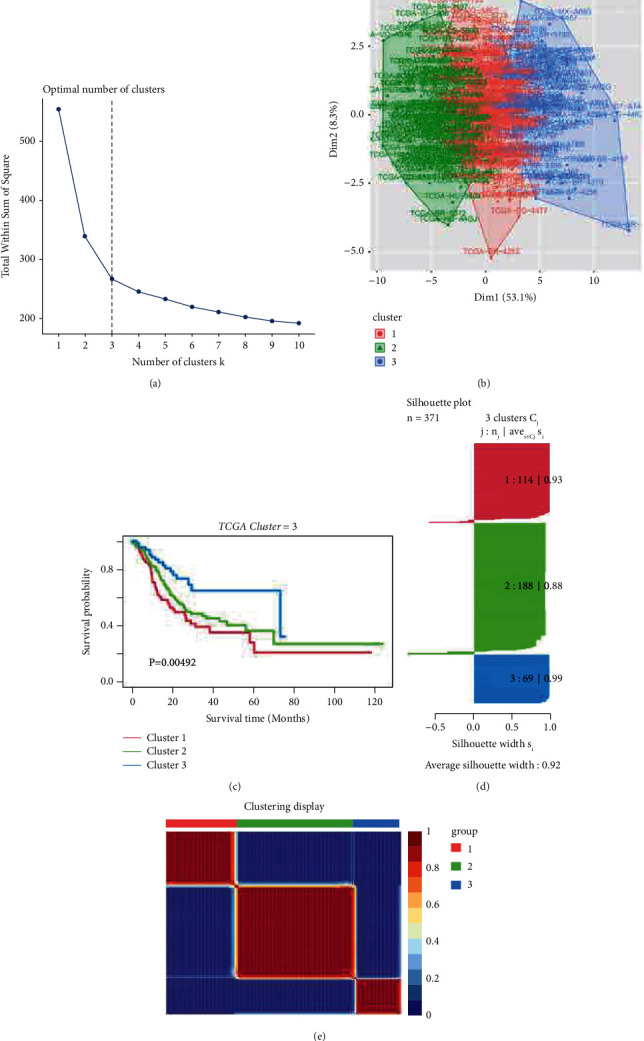
Establishment of subtypes of gastric cancer in TCGA cohort. (a) The optimal cutoff value of cluster was generated. (b) Visualization of cluster plot. (c) Survival patterns were drawn by survival analysis. (d) Silhouette plot was sketched. (e) Three clusters were identified.

First, raw RNA-sequence data (32 normal cases and 375 tumor cases) from TCGA and gene expression data (433 tumor cases) from the GEO database were downloaded for further validation. The flow diagram is shown in Figure 1. Immunologic gene sets from microarray gene profiling and hallmark gene sets (containing 4922 gene sets) from GSEA were used to calculate the enrichment score (ES) of each sample using the GSVA method. Differences were detected between normal tissues and gastric samples (Figure S1). Through reckoning, an ES value was obtained for each sample, and based on this, we established a Cox regression model to screen the data and then generated the optimal number of clusters by using *nbclust* and *factoextra* packages (Figures 2(a) and 2(b)). By executing *the cancersubtypes* package, gastric cancer was classified into three subtypes (Figures 2(d) and 2(e)), and significant differences were observed pertaining to OS in the three subtypes (Figure 2(c)). To validate the gastric cancer subtype generated from TCGA database, we collected and analyzed the data of GSE84437 downloaded from the GEO database. The accuracy of the cluster model was verified (Figure S2).

### 3.2. Relationship between Clinical Characteristics and Distinct Subtypes in Gastric Cancer

**Figure 3:**
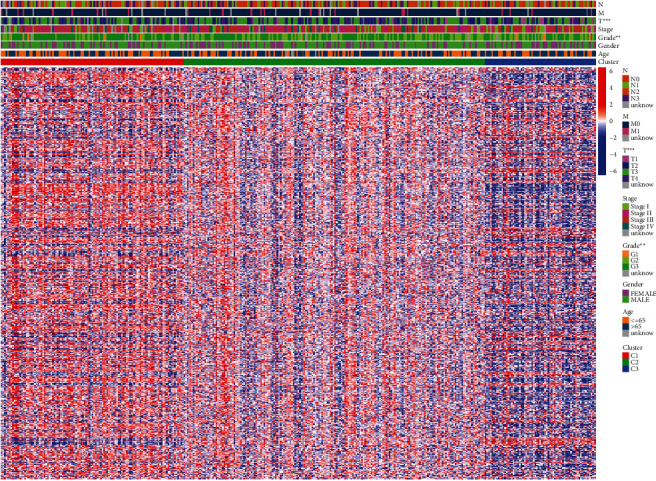
Heatmap of relationship between subtypes and clinical features.

**Figure 4:**
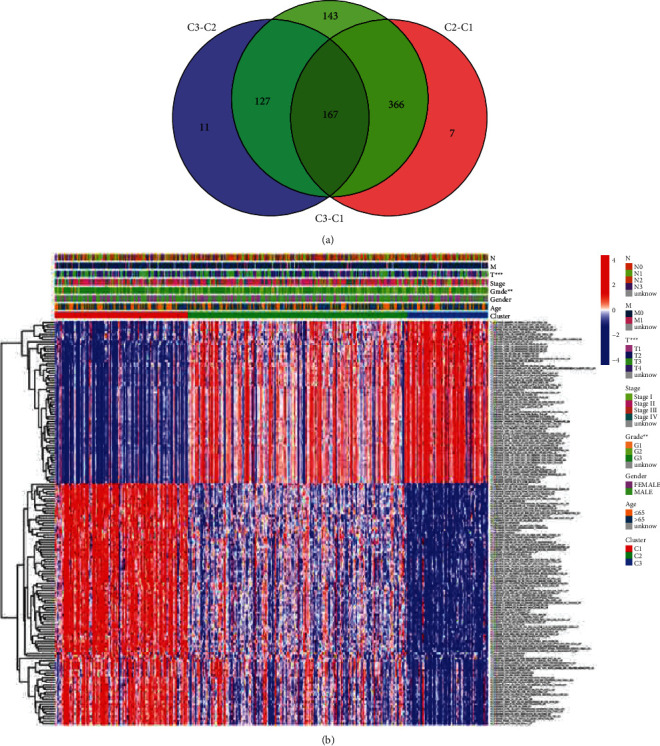
Generation of core gene sets among three clusters. (a) Veen map was drawn and identification of 167 gene sets by intersecting of three clusters. (b) Correlation between core gene sets and clinical features.

To explore the relationship between clinical features and gastric cancer subtypes, we performed a Chi-square test and found a significant difference between subtypes, *T* stage, and grade (Figure 3, Table S1). Afterwards, the variation in gene sets in the three subtypes was evaluated and calculated, and the adjusted P filter was set to 0.05. In total, 167 gene sets were detected (Figure 4(a)). Based on the results of the Venn diagram, we constructed the relationship between differentiated gene sets and clinical features and visualized them using the *pheatmap* package in the *R* language (Figure 4(b)).

### 3.3. Risk Model of Gene Sets Determined by LASSO Regression in Prognostic Setting

**Figure 5:**
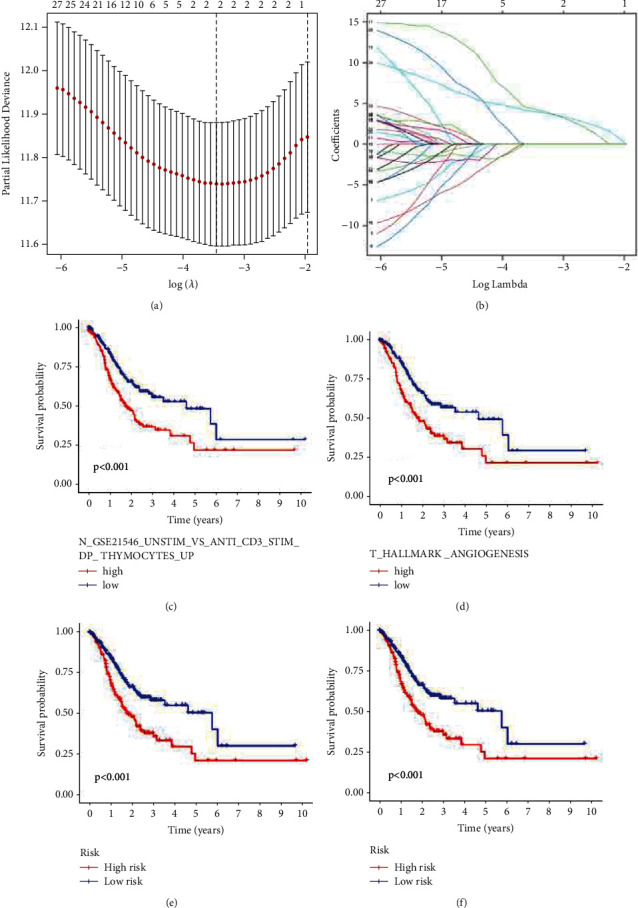
Identification of prognostic gene sets by Lasso regression model. (a) Optimal values of *λ* were shown. (b) Coefficient profiles of core gene sets were calculated. (c) Survival analysis of gene sets in normal tissues. (d) Survival analysis of gene sets in tumor tissues. (e) Prognostic value of LASSO regression risk model based on gene sets in TCGA cohort. (f) Validation of prognostic of risk model in GEO cohort.

Subsequently, a Cox regression model was established to acquire core gene sets related to the survival of patients with gastric cancer. The LASSO regression model was further constructed to identify the optimal cut-off point of GSVA and to split the patients into high- and low-risk groups based on the ES value of the gene sets (Figures 5(a) and 5(b)). Eventually, one normal gene set (N_GSE21546_UNSTIM_VS_ANTI_CD3_STIM_DP_ THYMOCYTES_UP) and one tumor gene set (T_HALLMARK _ANGIOGENESIS) were associated with the prognosis of patients with gastric cancer (Figures 5(c) and 5(d)). Patients in the low-risk group were more likely to have better prognosis (Figures 5(e) and 5(f)).

### 3.4. Functional Pathway Enrichment Analysis

**Figure 6:**
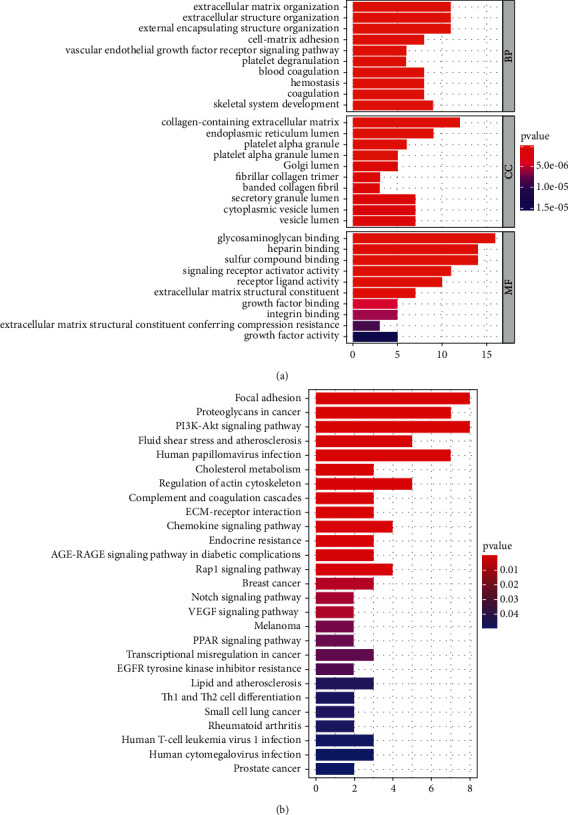
Functional analysis of genes from tumor gene set identified by LASSO method. (a) GO enrichment analysis. (b) KEGG enrichment analysis.

To further explore the function of the gene sets examined previously, we conducted GO and KEGG enrichment analyses of the genes extracted from the gene sets. Genes from the tumor gene set were associated with the vascular endothelial growth factor receptor signaling pathway, blood coagulation, focal adhesion, cholesterol metabolism, phosphatidylinositol 3-kinase/protein kinase B signaling pathway, regulation of actin cytoskeleton, and peroxisome proliferator-activated receptor signaling pathway (Figures 6(a) and 6(b)). All the identified factors contributed to the occurrence and development of gastric cancer. We then constructed a protein-protein interaction network using the STRING database (Figures S3(a) and S3(b)) (https://cn.string-db.org/). Thereafter, we imported the network into Cytoscape (version 3.9.0), analyzed the data using the molecular complex detection (MCODE) tool, and drew two core gene clusters for the tumor gene sets (Figures S3(c)–S3(f)). *VEGFA*, *TIMP1*, *FGFR1*, *PTK2*, and *COL3A1* are mainly related to oncogenic pathways, as mentioned above. *THBS1*, *FN1*, *SERPINE1*, *ZCCHC12*, *JPH3*, and *ELMOD1* are related enriched in P53 pathway, which may affect the prognosis of gastric cancer patients.

## 4. Discussion

Advances in sequence analysis technology have enabled the development of more targeted therapy options for patients with gastric cancer [21–23]. In this study, we calculated the enrichment score based on GSVA in each patient from TCGA and GEO databases and developed a novel classification using cancer subtype and a nonnegative matrix factorization method. Thereafter, we constructed a LASSO regression model to examine the gene sets, divided the patients into high- and low-risk groups, and examined the prognostic value of the gene sets identified above. In general, patients were categorized into three subtypes, which are clinically relevant and linked to different prognoses. Two gene sets encompassing a normal gene set and a tumor gene set were observed using LASSO regression model analysis. The genes extracted from the normal group were involved in the p53 signaling pathway and retinol metabolism, and genes from the tumor gene set were involved in various pathways promoting tumorigenesis in gastric cancer.

According to TCGA genomic features, various molecular classifications have been established to date [24–29], including microsatellite instability/mismatch repair (MSI/MMR) status, programmed death-ligand 1 (PD-L1) expression, tumor mutational burden (TMB) status, neurotrophic tropomyosin-related kinase fusions, and tumor Epstein–Barr virus, which revolutionizes the oncology therapy landscape. Malignant tumors with microsatellite instability-high (MSI-H) status or deficient MMR systems are prone to high-level antigens and linked to PD-L1 inhibitors. Although Kim et al. [30] detected a 10–22% incidence rate of gastric cancer in global districts, only 3% of patients with metastatic gastric cancer were certified to have MSI-H status. The paucity of the incidence rate of advanced gastric cancer delineated the limitation of the molecular features of MSI-H.

TMB refers to the total somatic nonsynonymous coding mutations per tumor exome and has been inferred to be correlated with the increased efficacy of immune checkpoint inhibitors. However, the optimal cutoff value for different types of tumors remains controversial. Rizvi et al. [31] claimed that patients with high TMB (>200 per megabase (Mb)) treated with pembrolizumab had better OS, while Hellmann et al. [32] concluded that lung cancer patients with higher TMB (≥10/Mb) treated with nivolumab and ipilimumab had better prognosis. Van Allen et al. [33] reported that neoantigen loads or tumor cytolytic expression might predict the response to ipilimumab in melanoma; however, further exploration is needed to determine the cut-off point. In addition, another study conducted by Ratti et al. [34]. reported that TMB outstripping 16/Mb was related to improved prognosis in patients with lung cancer. Yarchoan et al. [35] detected that the median TMB pertaining to increased response rates varied among various cancer subtypes. Recently, an exploratory analysis from Keynote-061 demonstrated that gastric cancer patients with a TMB greater than 10/Mb could benefit from pembrolizumab in the second-and later-line settings, even excluding patients with MSI-H status, which was inconsistent with the results of the study conducted by Greally et al. [36]; therefore, due to the complexity of TMB detection and heterogeneity of tumors, further studies are necessary to explore the predictive value of TMB in gastric cancer.

PD-L1 expression was determined by immunohistochemistry and has been confirmed to be related to increased efficacy of treatment in various cancer types. The objective response rate (ORR) of gastric cancer patients with positive PD-L1 expression treated by pembrolizumab was significantly superior to that of patients without PD-L1 expression. Furthermore, other programmed cell death-1 (PD-1) inhibitors (nivolumab/SHR-1210) have advantages over placebo in the second- or later-line setting, with nivolumab prolonging OS at 2-year follow-up and SHR-1210 increasing the ORR to 26.7% [37, 38]. In particular, combining nivolumab and 5-FU-based chemotherapy has been prioritized for advanced cancer in palliative therapy settings, with significant improvements in OS (hazard ratio 0.71, 98% confidence interval 0.59–0.86), and the therapeutic effect of these agents was not limited by PD-L1 expression [39, 40]. In conclusion, PD-L1 served as a prognostic and predictive marker for pembrolizumab but could not fit all PD-1/L1 inhibitors. Recently, the molecular subtypes mentioned above have provided innovative strategies for gastric cancer; however, concern remains. The emerging expansion of molecular profiling is necessary because of the heterogeneity of gastric cancer.

Various other molecular subtypes have been identified. Wang et al. [41]. discovered a novel subtype of *RHOA* mutations in diffuse-type gastric adenocarcinomas. Tan et al. [42, 43] proposed an intrinsic subtype for gastric cancer cell lines. Shah et al. [44] classified gene expression at different anatomic sites. The studies mentioned above focused on cancer tissues and ignored the functions of normal tissues. In this study, we classified three subtypes based on immunogenetic gene sets and hallmark gene sets, and changes in both tumor and nontumor tissues were analyzed. Two gene sets were identified: one from the normal tissue and one from the tumor tissue. Genes from the normal gene set were enriched in the P53 pathway and bladder cancer, which may promote the colonization of tumor cells. Moreover, genes from the tumor gene sets were involved in focal adhesion and regulation of the actin cytoskeleton, which is consistent with the results of a previous study. Hub genes containing *COL5A2*, *JAG1*, *TIMP1*, *FGFR1*, *PDFGA*, *VEGFA*, and *PTK2* were collected from the gene sets and were proven to be linked to the occurrence and development of gastric cancer.

Altogether, we sketched three subtypes encompassing two prognostic gene sets of gastric cancer based on tumor and nontumor data, highlighting the molecular features and significant role of nontumor genes in patients with cancer.

## Data Availability

All the data are available online.

## Abbreviations

5-fluorouracil: 5-FU
ES: Enrichment score
GEO: Gene expression omnibus
GO: Gene ontology
GSEA: Gene set enrichment analysis
GSVA: Gene set variation analysis
HER2: Human epidermal growth factor receptor 2
KEGG: Kyoto encyclopedia of genes and genomes
LASSO: Least absolute shrinkage and selection operator
MMR: Mismatch repair
MSI-H: Microsatellite instability-high
ORR: Objective response rate
OS: Overall survival
PD-1: Programmed cell death-1
PD-L1: Programmed death-ligand 1
STRING: Search tool for the retrieval of interacting genes/proteins
TCGA: The cancer genome atlas
TMB: Tumor mutational burden.

## Supplementary Materials

Supplementary Materials Figure S1. Visualization of enrichment score of gene sets in gastric cancer and adjacent nontumor tissues by gene set variation analysis. Figure S2. Establishment of subtypes of gastric cancer in the GEO cohort. (a). The optimal cutoff value of cluster was generated. (b). Visualization of cluster plot. (c). Survival patterns were drawn by survival analysis. (d) Silhouette plot was sketched. (e) Three clusters were identified. Figure S3. The protein-protein interaction network of genes in tumor gene set (a), (c), (e) and in normal gene set (b), (d), (f). Table S1. Correlation between clinical features and cancer subtypes.
