# Supplementary material for: Delineating Molecular Subtypes through Gene Set Variation Analysis Confers Therapeutic and Prognostic Capability in Gastric Cancer
Source: Can J Gastroenterol Hepatol. 2022 Jul 15;2022:5415758. doi: 10.1155/2022/5415758 (PMC9307400; doi:10.1155/2022/5415758)
Supplement: Supplementary Materials — Figure S1. Visualization of enrichment score of gene sets in gastric cancer and adjacent nontumor tissues by gene set variation analysis. Figure S2. Establishment of subtypes of gastric cancer in the GEO cohort. (a). The optimal cutoff value of cluster was generated. (b). Visualization of cluster plot. (c). Survival patterns were drawn by survival analysis. (d) Silhouette plot was sketched. (e) Three clusters were identified. Figure S3. The protein-protein interaction network of genes in tumor gene set (a), (c), (e) and in normal gene set (b), (d), (f). Table S1. Correlation between clinical features and cancer subtypes. [file 5415758.f1.docx]

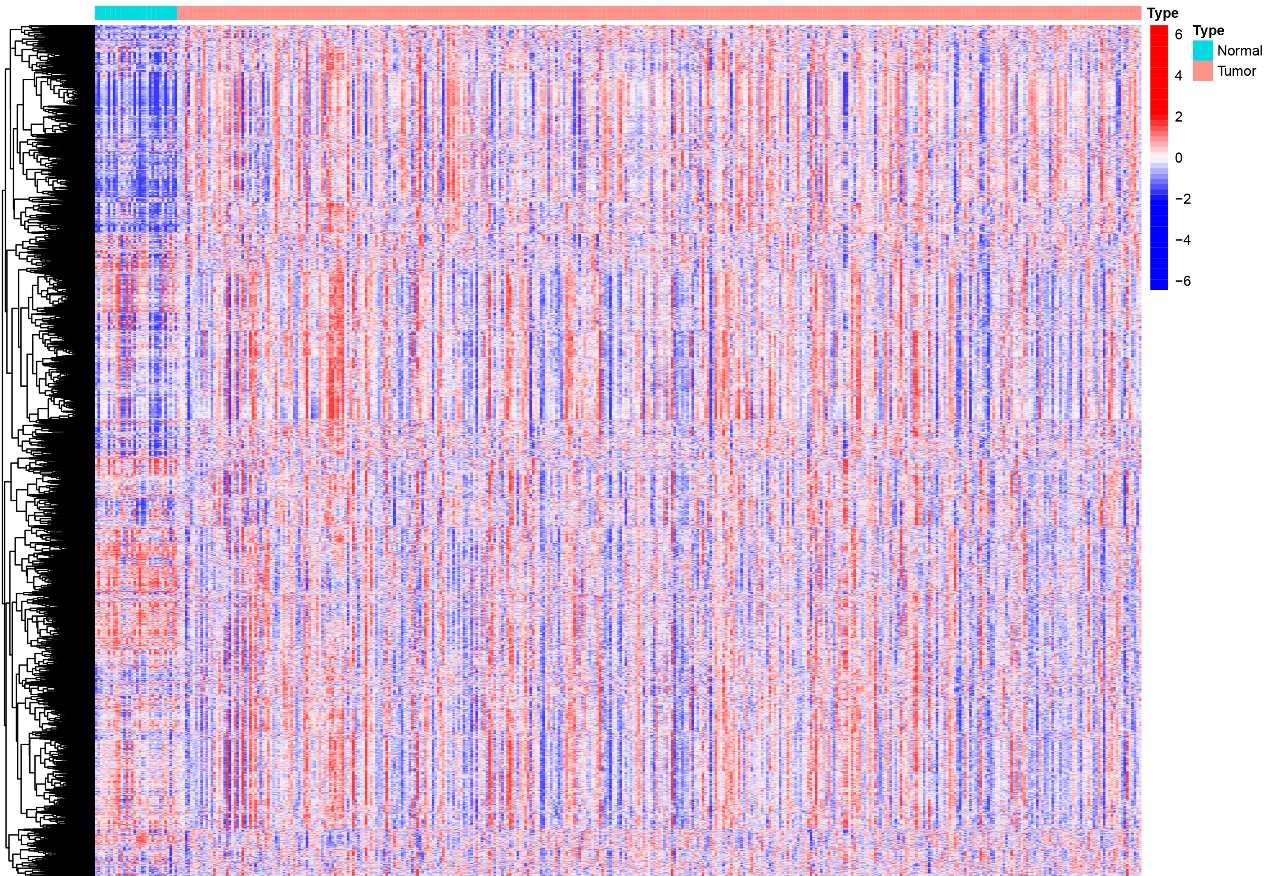


Figure S1. Visualization of enrichment score of gene sets in gastric cancer and adjacent non tumor tissues by gene set variation analysis.


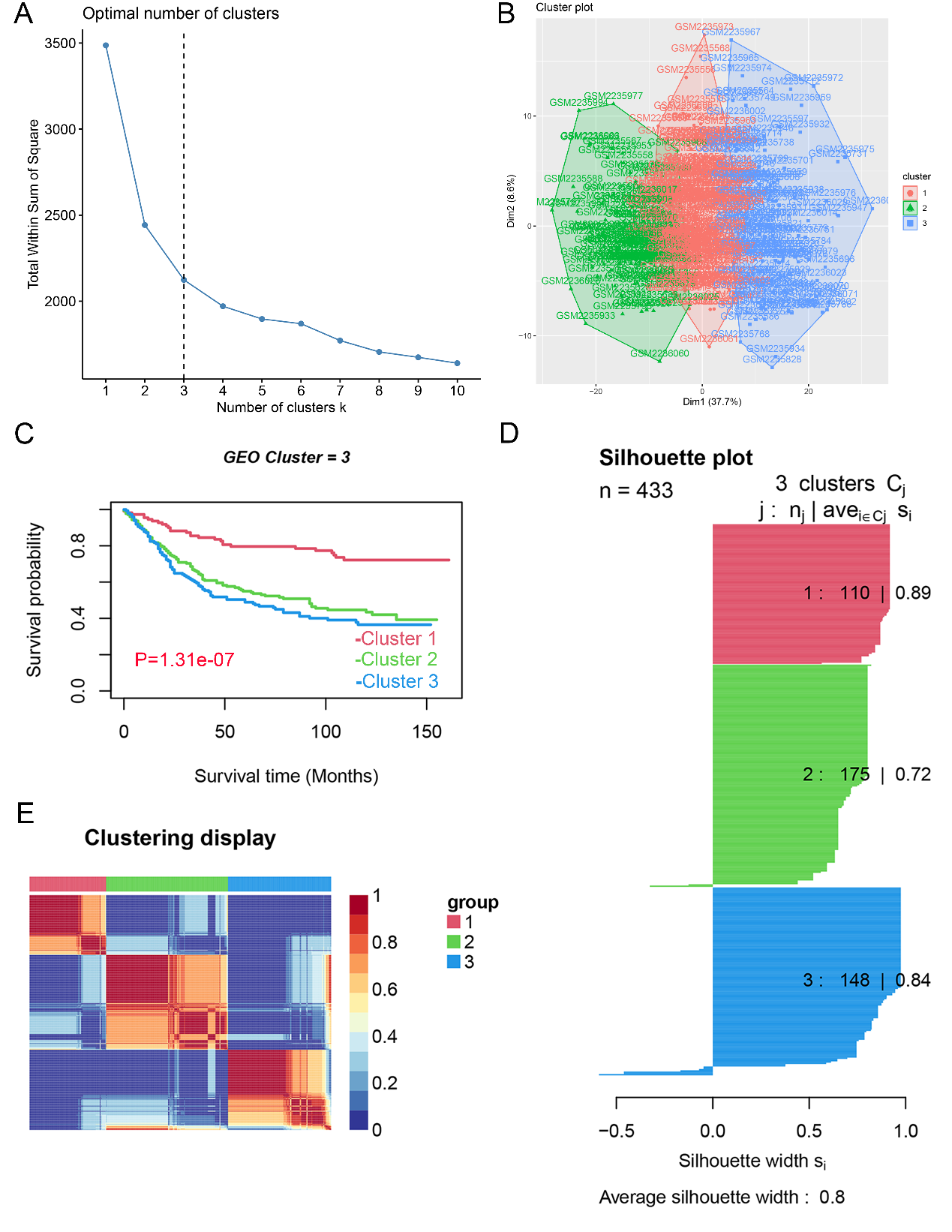


Figure S2. Establishment of subtypes of gastric cancer in GEO cohort. A. the optimal cutoff value of cluster was generated. B. visualization of cluster plot. C. Survival patterns were drawn by survival analysis. D. Silhouette plot was sketched. E. three clusters were identified.


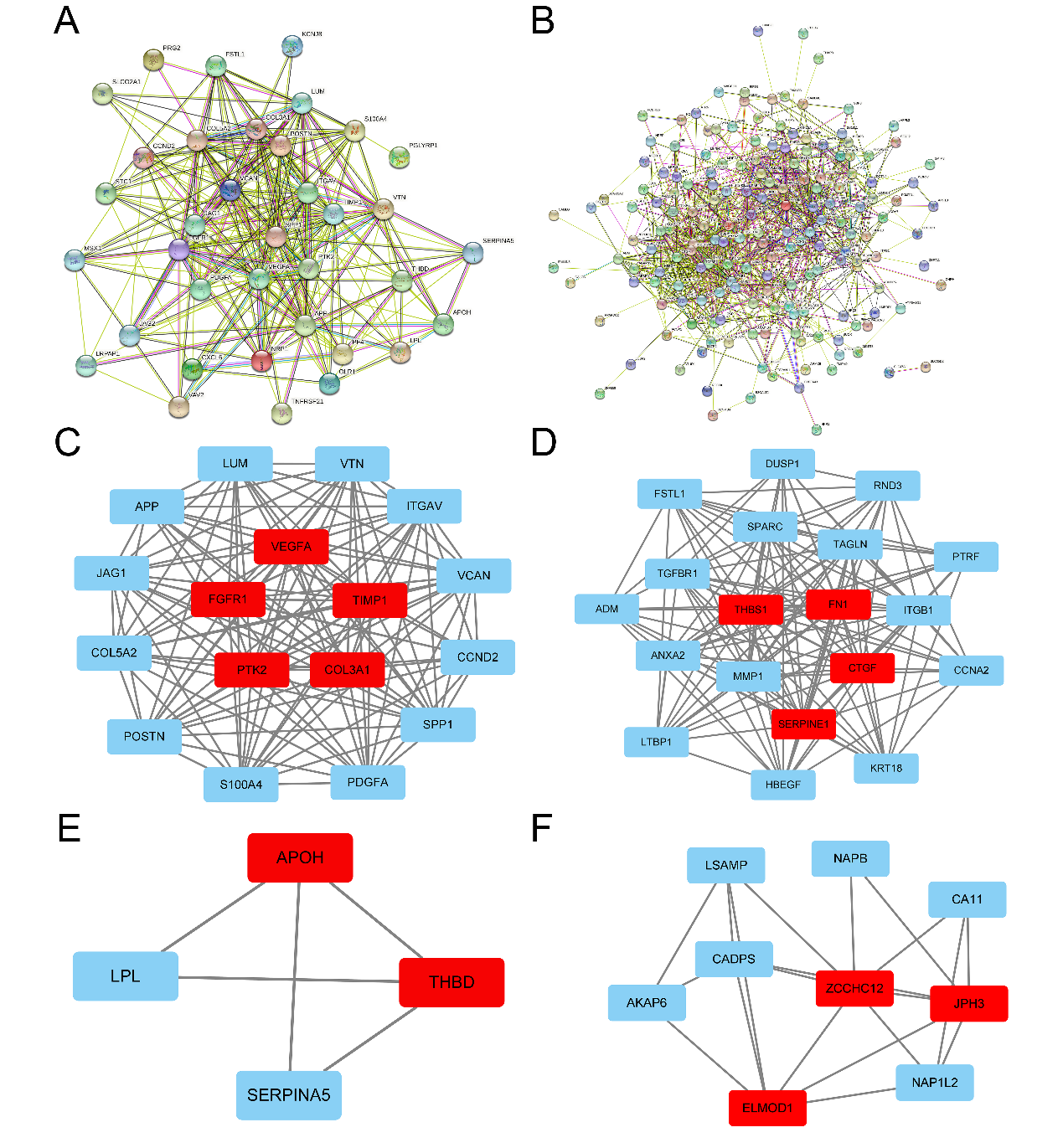


Figure S3. The protein-protein interaction network of genes in tumor gene set(A,C,E) and in normal gene set(B,D,F).

Table S1. Correlation between clinical features and cancer subtypes.

| Covariates | Cluster | Total | C1 | C2 | C3 | Pvalue |
| --- | --- | --- | --- | --- | --- | --- |
| Age | <=65 | 163(43.94%) | 58(50.88%) | 78(41.49%) | 27(39.13%) | 0.2135 |
| Age | >65 | 205(55.26%) | 56(49.12%) | 107(56.91%) | 42(60.87%) |  |
| Age | unknow | 3(0.81%) | 0(0%) | 3(1.6%) | 0(0%) |  |
| Gender | FEMALE | 133(35.85%) | 39(34.21%) | 64(34.04%) | 30(43.48%) | 0.3419 |
| Gender | MALE | 238(64.15%) | 75(65.79%) | 124(65.96%) | 39(56.52%) |  |
| Grade | G1 | 10(2.7%) | 5(4.39%) | 1(0.53%) | 4(5.8%) | 0.001 |
| Grade | G2 | 134(36.12%) | 26(22.81%) | 81(43.09%) | 27(39.13%) |  |
| Grade | G3 | 218(58.76%) | 79(69.3%) | 103(54.79%) | 36(52.17%) |  |
| Grade | unknow | 9(2.43%) | 4(3.51%) | 3(1.6%) | 2(2.9%) |  |
| Stage | Stage I | 50(13.48%) | 11(9.65%) | 21(11.17%) | 18(26.09%) | 0.0539 |
| Stage | Stage II | 111(29.92%) | 32(28.07%) | 62(32.98%) | 17(24.64%) |  |
| Stage | Stage III | 149(40.16%) | 47(41.23%) | 76(40.43%) | 26(37.68%) |  |
| Stage | Stage IV | 38(10.24%) | 10(8.77%) | 23(12.23%) | 5(7.25%) |  |
| Stage | unknow | 23(6.2%) | 14(12.28%) | 6(3.19%) | 3(4.35%) |  |
| T | T1 | 18(4.85%) | 1(0.88%) | 6(3.19%) | 11(15.94%) | 9.00E-04 |
| T | T2 | 78(21.02%) | 24(21.05%) | 41(21.81%) | 13(18.84%) |  |
| T | T3 | 167(45.01%) | 50(43.86%) | 89(47.34%) | 28(40.58%) |  |
| T | T4 | 100(26.95%) | 31(27.19%) | 52(27.66%) | 17(24.64%) |  |
| T | unknow | 8(2.16%) | 8(7.02%) | 0(0%) | 0(0%) |  |
| M | M0 | 328(88.41%) | 100(87.72%) | 165(87.77%) | 63(91.3%) | 0.9247 |
| M | M1 | 25(6.74%) | 8(7.02%) | 13(6.91%) | 4(5.8%) |  |
| M | unknow | 18(4.85%) | 6(5.26%) | 10(5.32%) | 2(2.9%) |  |
| N | N0 | 108(29.11%) | 27(23.68%) | 56(29.79%) | 25(36.23%) | 0.5911 |
| N | N1 | 97(26.15%) | 30(26.32%) | 48(25.53%) | 19(27.54%) |  |
| N | N2 | 74(19.95%) | 19(16.67%) | 43(22.87%) | 12(17.39%) |  |
| N | N3 | 74(19.95%) | 26(22.81%) | 37(19.68%) | 11(15.94%) |  |
| N | unknow | 18(4.85%) | 12(10.53%) | 4(2.13%) | 2(2.9%) |  |
